# UVB (290–315 nm) inactivation of the SARS CoV-2 virus as a function of the standard UV index

**DOI:** 10.1007/s11869-021-01099-3

**Published:** 2021-11-04

**Authors:** Jay Herman, Rubén D. Piacentini

**Affiliations:** 1 University of Maryland Baltimore County JCET, Baltimore, MD USA; 2 Institute of Physics Rosario, CONICET - National University of Rosario and Technological Institute of Design and Innovation, Faculty of Exact Sciences, Engineering and Surveying, National University of Rosario, Rosario, Argentina

**Keywords:** COVID-19, SARS-CoV-2, Inactivation, Radiative transfer, Ozone, UVB, UVC, UV Index

## Abstract

The inactivation time for the SARS CoV-2 virus, mostly by a portion of UVB spectrum (290–315 nm) in sunlight, has been estimated using radiative transfer calculations and a relative wavelength sensitivity virus inactivation action spectrum A_LS_. The action spectrum is adjusted for the SARS CoV-2 virus using a derived UV dose D_90_ = 3.2 J/m^2^ for 90% inactivation to match laboratory results for the inactivation of SARS CoV-2 virus droplets on steel mesh. Estimation of the time for 90% inactivation T_90_ at a specific geographic location can be simplified using the commonly published or calculated UV index (UVI). The use of UVI has the advantage that information on the amount of ozone, the site altitude, and the degree of cloud cover are built into the published UVI calculation. Simple power-law T_90_(UVI) = *a* UVI^*b*^ fitting equations are derived that provide estimates of T_90_(UVI) for 270 specific locations. Using the results from the 270 locations, a generalized latitude θ dependence is presented for the coefficients *a*(θ) and *b*(θ) that enables T_90_(θ, UVI) to be estimated for 60°S ≤ θ ≤ 60°N and for noon and 2 h around local solar noon.

**Supplementary Information:**

The online version contains supplementary material available at 10.1007/s11869-021-01099-3.

## Introduction

The 90% inactivation time T_90_ of the SARS CoV-2 virus by 254 nm UVC light has been estimated from laboratory experiments (Anderson et al. 2013; Bedell et al. 2016; Heßling et al. 2020; Lytle and Sagripanti 2005; Sagripanti and Lytle 2020; Kowalski et al. 2009; Kowalski 2009) along with significant but reduced inactivation rates for exposure to 290 to 315 nm UVB light (Eisenstark 1987; Nelson et al. 2018; Ratnesar-Shumate et al. 2020; Herman et al. 2020a). This paper will briefly examine the use of the UV index (UVI = erythemal irradiance in mW/m^2^ divided by 25 mW/m^2^) to estimate T_90_(UVI) for the latitude range 60°S ≤ θ ≤ 60°N. The advantage of using the UVI is that the index is forecast and published daily for many locations around the world (Heckman et al. 2019), so that T_90_(UVI) is a useful tool to estimate ultraviolet germicidal irradiance (UVGI) viral inactivation in the UVB range. In addition, the UVI measurements or estimates already contain information on ozone amount, aerosol amount, cloud cover, and altitude for each site needed for the estimate of T_90 _(see ESM Electronic Supplemental Material or https://avdc.gsfc.nasa.gov/pub/DSCOVR/JayHerman/CoV_vs_UVI/ ).

## Inactivation time T_90_(UVI) of SARS CoV-2 by UVGI

In the paper by Herman et al. (2020a), T_90_ was calculated from a derived relative wavelength sensitivity action spectrum for virus inactivation by UVC and UVB exposure (Lytle and Sagripanti 2005). This action spectrum A_LS_ (λ was used to derive the equivalent 254 nm inactivation parameter D_90_ = 3.2 J/m^2^ (Herman et al. 2020a) that gives approximately the same results as the laboratory inactivation measurements for SARS-CoV-2 on steel mesh exposed to simulated sunlight (Ratnesar-Shumate et al. 2020). The amount of irradiance at the Earth’s surface F_O_(λ) (mW/m^2^) needed to estimate T_90_ in the critical 290 to 315 UVB range was calculated for over 270 locations (Herman et al. 2020b) with their elevations plus the daily effects of cloud and aerosol cover, ozone amounts all obtained from the OMI (Ozone Monitoring Instrument) satellite data record (Schenkeveld et al. 2017). The key equation linking the laboratory results with inactivation time is given by Eqs. 1 and 2.

1 $$\frac{\mathbf{N}}{{\mathbf{N}}_{\mathbf{0}}}={\mathbf{e}}^{-\mathbf{kD}}$$

The dose D = D_90_ is a time accumulated irradiance exposure (J/m^2^) such that the survival fraction N/N_0_ = 0.1, where N = the number of virus particles left after exposure D (J/m^2^) to UVB starting with N_O_ particles.

T_90_ is then calculated from

2 $$ {\mathrm{T}}_{90}=\frac{{\mathrm{D}}_{90}}{\int_{290}^{320}{\mathrm{A}}_{\mathrm{LS}}\left(\uplambda \right){\mathrm{F}}_{\mathrm{o}}\left(\uplambda \right)\mathrm{d}\uplambda}\left(\mathrm{Seconds}\right) $$

F_O_(λ) and F_O_(λ) A_LS_(λ) are calculated from the publicly available tropospheric ultraviolet and visible (TUV) scalar radiative transfer code (Madronich 1993, 1995; Herman et al. 2020b) https://www2.acom.ucar.edu/modeling/tuv-download for every day of each year for 16 years from 2005 to 2020. Recent inactivation measurements of SARS CoV-2 exposed to sunlight for virus containing droplets onto a stainless-steel wire mesh (Ratnesar-Shumate et al. 2020) led to an estimate of D_90_ = 3.2 J/m^2^ (Herman et al. 2020a), which is used for the analysis presented here. If different values for D_90_ are used, the results are proportionately scaled. The time *t* for decreased survival fraction can easily be estimated because of the exponential relationship in Eq. 1. That is, if N/N_O_ = 0.001, then T_99.9_ = 3T_90_.

**Fig. 1:**
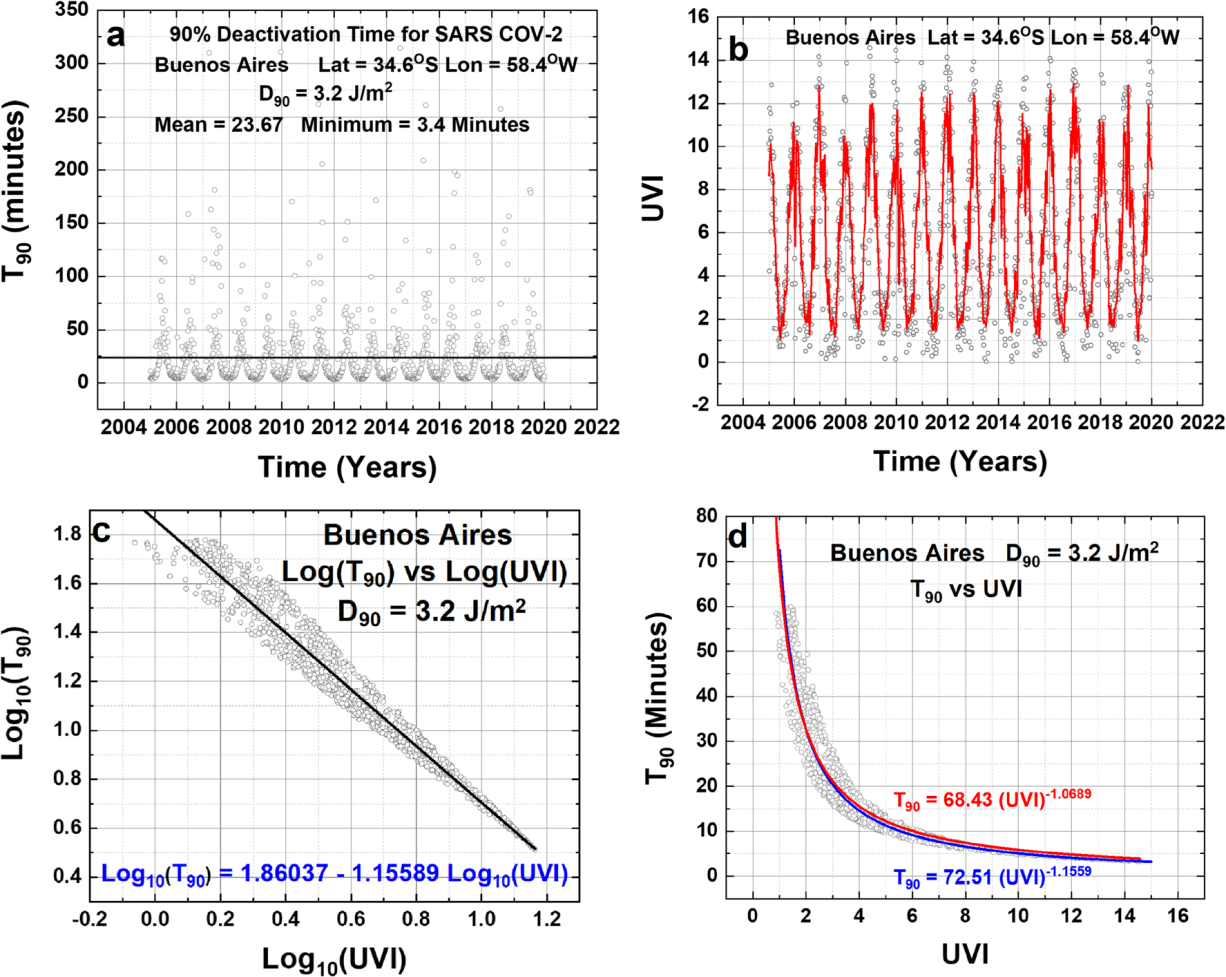
**a** Calculated T90 time series for Buenos Aires, Argentina. **b** UVI time series. **c** Log-Log scale plot of T90 vs UVI. **d** Linear scale plot of T90 vs UVI containing the direct fit (red line) and the Log-Log fit (blue line). The mean inactivation time is 23.67 min, and the minimum is 3.4 min.

**Fig. 2:**
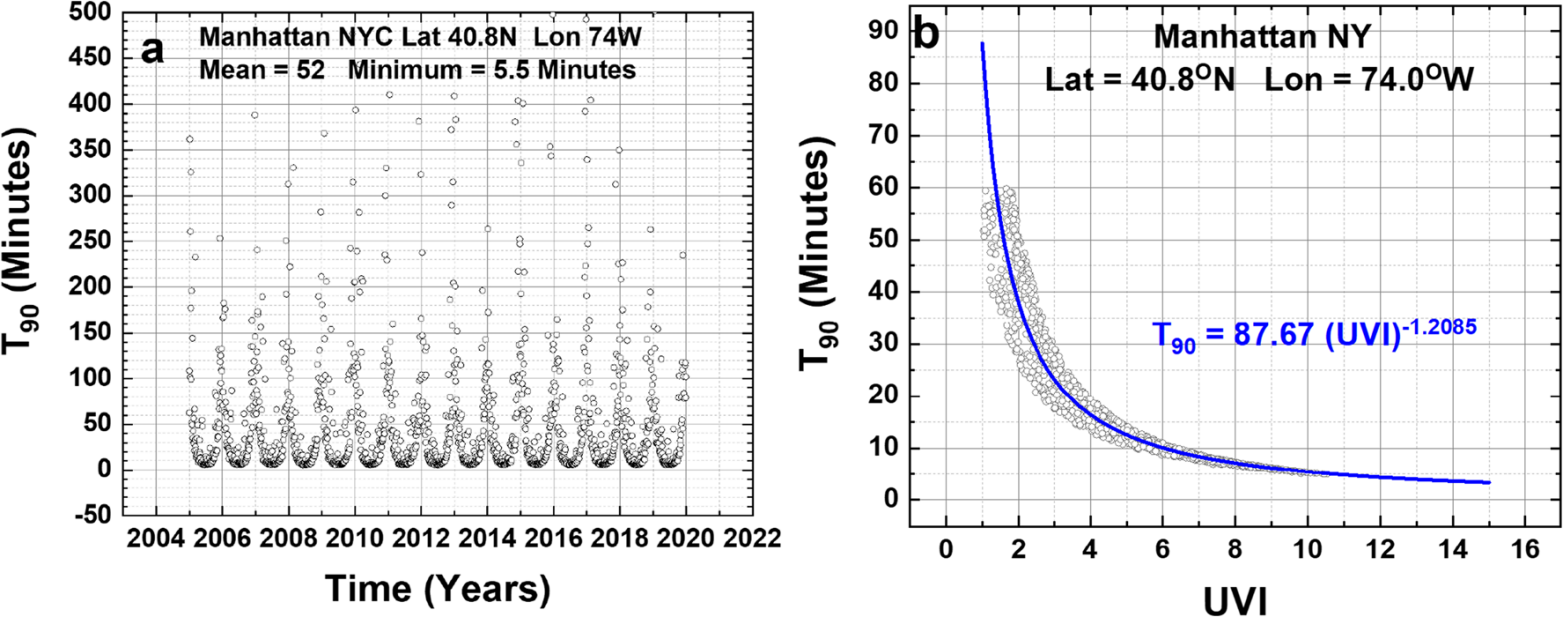
**a** T_90_ time series. **b** T_90_ vs UVI for Manhattan, New York City, for D_90_ = 3.2 J/m^2^. Mean = 52 min with a summer minimum of 5.5 min. **b** Power law fit to T_90_(UVI)

**Fig. 3:**
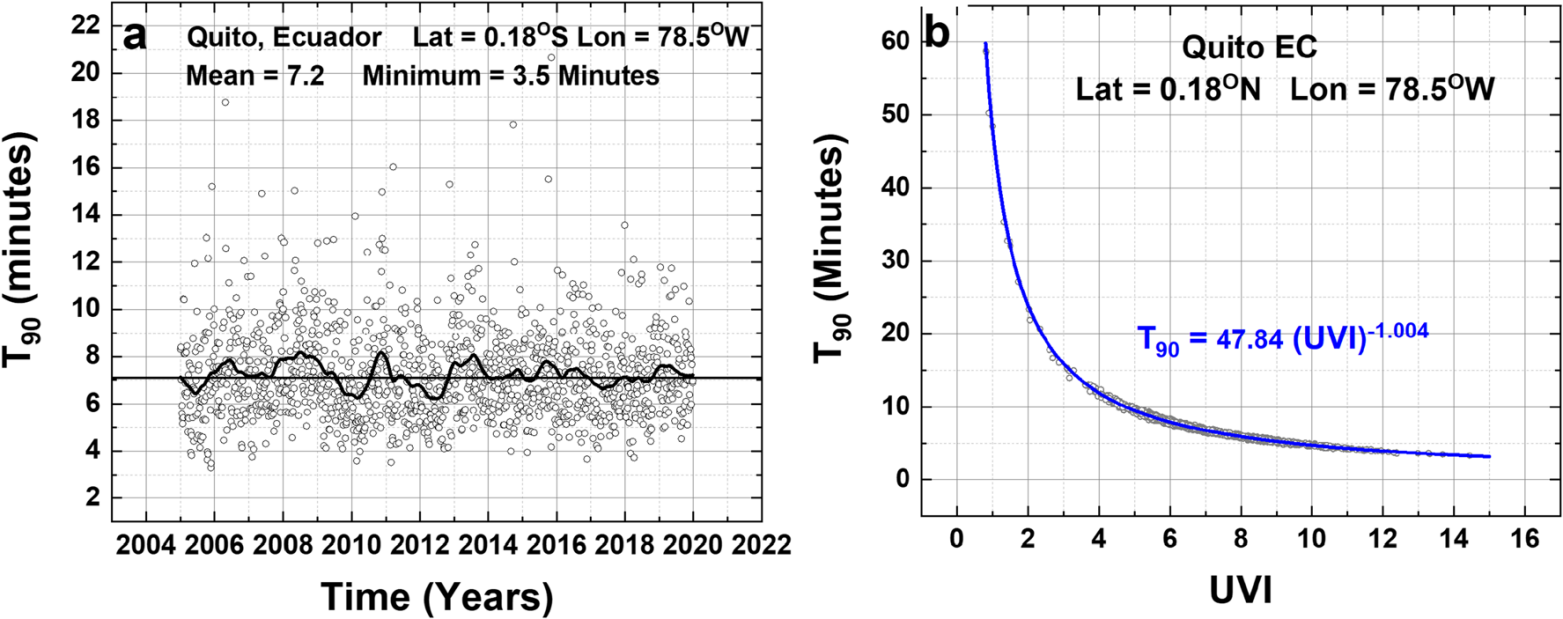
**a** T_90_ time series. **b** T_90_ vs UVI for Quito, Ecuador, for D_90_ = 3.2 J/m^2^. Mean = 7.2 min with an exceptionally clear-sky minimum of 3.5 min that occurs occasionally during the 16 years. The black line is a Loess (0.2) fit to the T_90_ data. **b** Power law fit to T_90_(UVI) data

Applications of Eqs. 1 and 2 for three specific sites are shown in Figs. 1 to 3. Figure 1 outlines the method of finding the relationship between the calculated T_90_ and UVI time series. The T_90_ time series for Buenos Aires, Argentina (Fig. 1a) shows the seasonal dependence with a minimum in summer and a maximum in winter in a range from 3.4 to 50 min for most days of the year. Values can exceed 50 min when it is exceptionally cloudy. The strong seasonal dependence of the UVI (Fig. 1b) anti-correlates well with T_90_ so that high values of UVI correspond to low values of T_90_. When the T_90_ is plotted against UVI (Figs. 1c and d), the resulting distribution of points closely resembles a power-law function (Eq. 3).

3 $${\mathbf{T}}_{\mathbf{90}}=\mathbf{a}\left(\mathbf{UVI}\right)^b$$

The coefficients *a* and *b* in Eq. 3 can be derived using either of two methods. First, is a fitting error minimization procedure applied directly to the data points shown in Fig. 1d (red) or, second, derived from a linear fit (blue, Eq. 4) shown in the Log-Log plot of Fig. 1c. The linear fit for the blue curve Log-Log fit yields a better result passing closer through the center of the data points for UVI > 2 than the direct minimization fit that is above the data points (Fig. 1d).

4 $${\mathbf{T}}_{\mathbf{90}-\mathbf{BA}}=\mathbf{72.51}\ {\left(\mathbf{UVI}\right)}^{-\mathbf{1.1559}}\space\mathbf{(Log}-\mathbf{Log}\ \mathbf{fit)}$$

A second example T_90-MAN_ is shown for Manhattan, New York City (Latitude 40.8°N), where the fit from the Log-Log linear fit method (Eq. 5) also produces better results for UVI > 2 than the direct-fit method (Fig. 2).

5 $${\mathbf{T}}_{\mathbf{90}-\mathbf{Man}}=\mathbf{87.67}\ {\left(\mathbf{UVI}\right)}^{-\mathbf{1.2085}}\space\mathbf{(Log}-\mathbf{Log}\ \mathbf{fit)}$$

A third example is shown for equatorial data from Quito, Ecuador (latitude 0.18°S) where there is almost no seasonal dependence in the T_90_ time series (Fig. 3a), but with a weak biennial signal suggested by the black-line Loess (0.2) fit, (Cleveland 1979; Cleveland 1981). For this case, the direct fit and the Log-Log linear fit are indistinguishable. The time series and correlation plots presented from these 3 sites are typical for all 270 sites (220 land sites and 50 ocean sites) considered (ESM Appendix Table 1). The Pacific Ocean sites are located along lines of longitude −60° ≤ θ ≤ 60° at 179°W and the Atlantic Ocean sites at 30°W. Another 25 land sites (labelled SH_NH) are located along 60°W from −60° ≤ θ ≤ 60°. The remaining 195 land sites are mostly large cities, −60° ≤ θ ≤ 60°.

6 $${\mathbf{T}}_{\mathbf{90}-\mathbf{Quito}}=\mathbf{47.84}\ {\left(\mathbf{UVI}\right)}^{-\mathbf{1.004}}\space\mathbf{(Log}-\mathbf{Log}\ \mathbf{fit)}$$

**Fig. 4:**
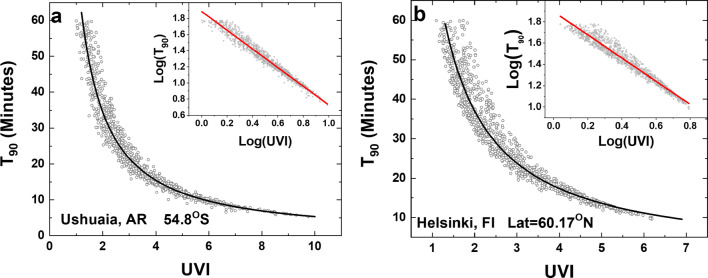
Two high latitude sites. **a** Ushuaia, Argentina, and **b** Helsinki, Finland. The solid lines are from the fitting parameters *a* and *b* in  ESM Appendix Table 1. T_90-Ush_ = 76.89 (UVI)^−1.1578^ and T_90-Hel_ = 78.66 (UVI)^−1.0890^

Figure 4 shows two high latitude sites that do not have many values of high UVI. Helsinki, Finland, at 60.17°N rarely has UVI > 6 and Ushuaia, Argentina, 54.8°S only has a few points greater than UVI = 8 that are associated with exceptionally low ozone values when the Antarctic ozone hole passes over Ushuaia.

**Fig. 5:**
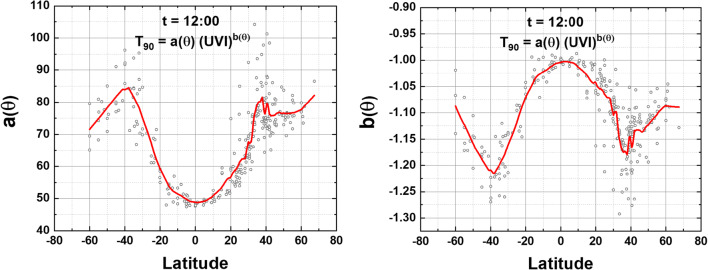
Graph of fitting coefficients *a*(θ) and *b*(θ) obtained from each location in ESM Appendix Table 1. The solid lines are a Loess (0.1) fit to *a*(θ) and *b*(θ) for local solar time *t* = 12:00

**Fig. 6:**
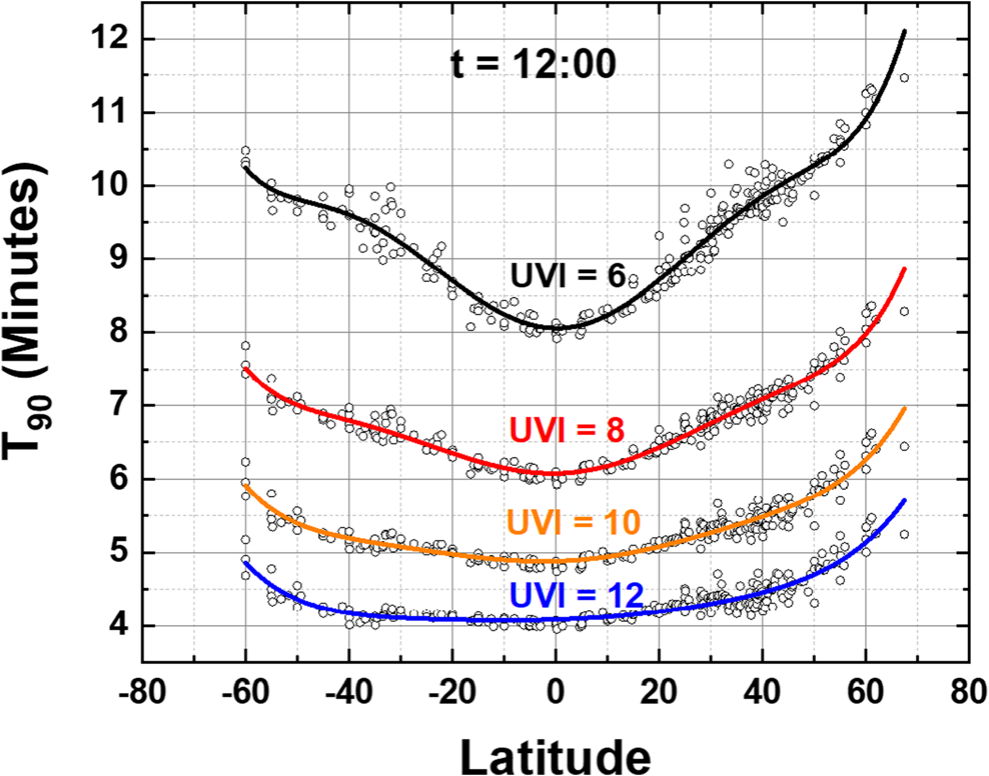
Application of ESM Appendix Table 2 (solid lines) with Eq. 7 to estimate T_90_(θ) for UVI = 6, 8, 10, and 12 for local solar time *t* = 12:00. The circles are sites from ESM Appendix Table 1

 ESM Table S1 shows the results for the coefficients *a* and *b* (Eq. 3) based on the Log-Log linear fit method for the 270 selected locations 60°S to 60°N and various longitudes covering the Earth including the Atlantic and Pacific Ocean locations. When sorted by latitude, there is a clear latitudinal dependence of T_90_(θ) vs UVI(θ) and in *a*(θ) and *b*(θ) (Figs. 5 and 6).

The coefficients *a*(θ) and *b*(θ) from ESM Appendix Table 1 are plotted vs latitude −60° ≤ θ ≤ 67° for solar time *t* = 12:00 (Fig. 5). The shape of the *a*(θ) and *b*(θ) curves at θ > 40° N and S have no physical meaning but are an artifact of fitting UVI data with increased scatter from higher ozone values and increased amounts of cloud cover compared to sites at lower latitudes.

**Fig. 7:**
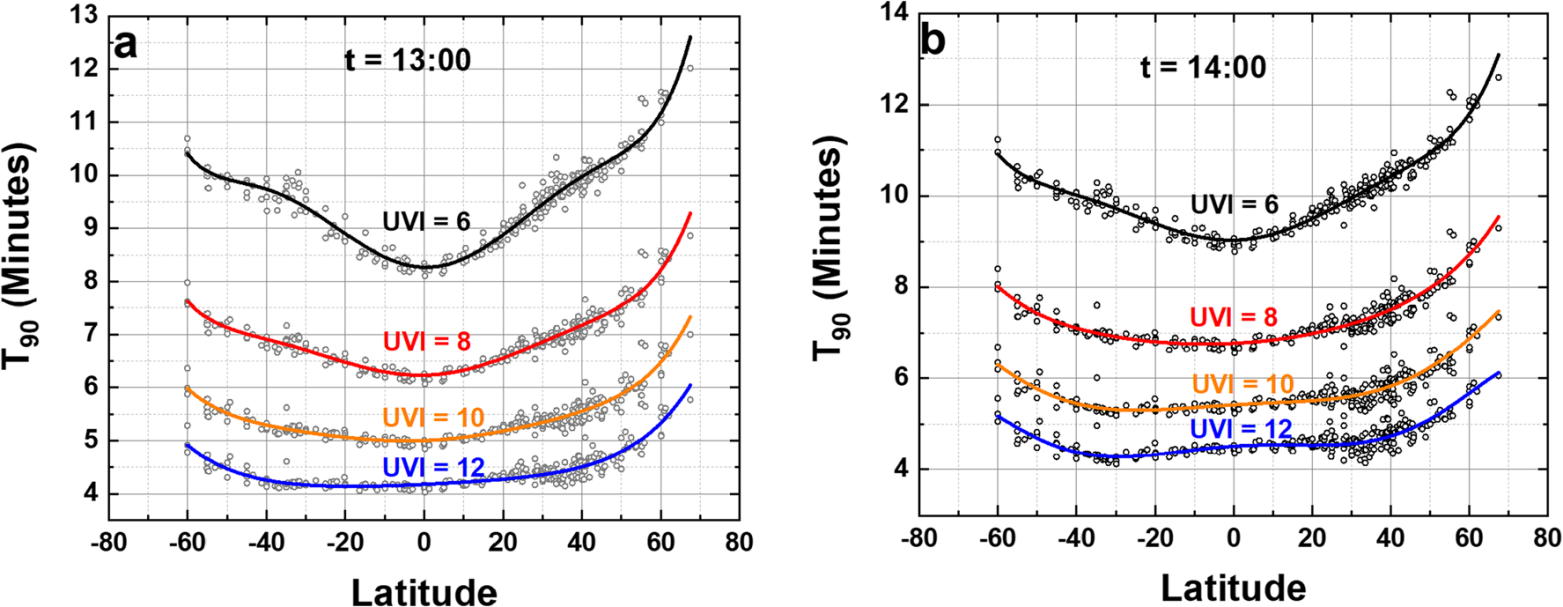
Same as Fig. 6 except for different times of day, *t* = 13:00 to 14:00 local solar time. For the solid lines, see ESM Appendix Tables 3 and 4, for the fitting coefficients for the solid lines

The values of T_90_ obtained from Eq. 7 are longitude averages of the considered locations from ESM Appendix Table 1 that give an estimate of the inactivation time in minutes for a given value of UVI. If the UVI is only provided for *t* = 12:00 noon conditions, estimates for other times of the day can be obtained (Herman et al. 2020a) if the atmospheric conditions are approximately the same as local noon (see Figs. 6 and 7).

7 $${\mathbf{T}}_{\mathbf{90}}\left(\boldsymbol{\uptheta} \right)=\mathbf{a}\left(\boldsymbol{\uptheta} \right)\ {\left(\mathbf{UVI}\right)}^{\mathbf{b}\left(\boldsymbol{\uptheta} \right)}$$

Figure 6 shows the results of using Eq. 7 to estimate T_90_ as a function of latitude θ (degrees) for specified values of UVI estimated at 12:00 local solar time. T_90_(θ) calculated as in Fig. 6 differs from specific site values because the fitting coefficients, *a*(θ) and *b*(θ), are a least-squares longitude-latitude fit from the 60°S to 67°N tabulated sites (Fig. 5) with the solid lines a Loess (0.1) fit to the data points.

The different latitude dependent shapes of *a*(θ), *b*(θ), and T_90_(θ, UVI) for different solar times *t* are partly an effect of the solar zenith angle SZA and O_3_ amounts and its differential effect on A_LS_(λ)F_0_(λ), 290 < λ < 315 nm, compared to the action spectrum for erythemal irradiance A_ery_(λ)F_0_(λ), 300 < λ < 400 nm (McKinlay and Diffey 1987; Webb et al. 2011). There are different atmospheric absorption amounts for short wavelength UVB (290–315 nm) compared to UVI (300–400 nm) penetrating to the Earth’s surface. The latitude dependence of T_90_ and UVI are different from equatorial zone latitudes, because of smaller solar zenith angle SZA and lower equatorial zone total column ozone TCO, compared to higher latitudes because of increasing TCO and SZA. In addition, Figs. 6 and 7 combine different seasons, altitudes, and different cloud conditions in each T_90_(θ, UVI) curve, since they are derived from a longitude average of entries in ESM Appendix Table 1. For a particular location, an estimate of T_90_(UVI) can be found using the specific *a*(θ) and *b*(θ) from ESM Appendix Table 1 or using one nearby the desired location.

Figure 7 shows the effect of different local solar times (13:00 and 14:00) on the value of T_90_(UVI) assuming that the atmosphere is the same as at noon. The graphs also apply at 10:00 and 11:00 as a function of time from local solar noon.

At the equator for a site with UVI = 10 at noon T_90_ = 5 min. Later in the day, say 14:00 h, if UVI decreases to UVI = 6, then and T_90_ = 9 min. UVI = 6 would be a clear bright-sun day in March or September at low altitude mid-latitude sites such as in Washington, DC, Rome, Italy, or Lauder, NZ.

## Summary

A method for estimating the 90% inactivation time T_90_(UVI) for SARS-CoV-2 virus from measured or calculated UV index UVI has been derived for 270 specific sites (ESM Appendix Table 1) in terms of power law T_90_(UVI, θ) = *a*(θ) (UVI)^*b*(θ)^ approximations. The Log-Log plot preferred method for determining the coefficients *a*(θ) and *b*(θ) is presented for three mid-latitude sites, Buenos Aires (34.6°S, 54.4°W), Manhattan, New York City (40.8°N, 74°W), an equatorial site Quito, Ecuador (0.18°S, 78.5°W), and two high latitude sites, Helsinki (60.2°N, 24.9°E) and Ushuaia, Argentina (50.80°S, 68.30°W). All five sites have T_90_anti-correlated with UVI, and correlation plots of T_90_ vs UVI suggest that a power-law fit is appropriate over a wide range of latitudes. A summary of the latitude θ dependence of the coefficients *a*(θ), *b*(θ), and T_90_(θ) is presented based on the fitting results for 270 specific sites in ESM Appendix Table 1. T_90_(θ, UVI, *t*) is shown for 4 different values of UVI (UVI = 6, 8, 10, and 12) typical of summer *t* = 12:00 values for mid- and low-latitude sites. T_90_(θ, UVI, *t*) was estimated for additional solar times, 13:00 and 14:00 h assuming the atmosphere is the same as at 12:00. T_90_(θ, UVI, *t*) for morning values are the same as afternoon values relative to their time difference from noon 10:00 and 11:00 h. Intermediate values of T_90_(θ, UVI, *t*) can easily be estimated. The graphs and ESM Appendix Table 1 provide a quick method to estimate T_90_, if the commonly published UVI is known or calculated.

## Supplementary Information

ESM 1  (DOCX 78 kb)

## Data Availability

All data used in this study are available in stated public archives, listed references, or included explicitly in the study. OMI data used in this study are available from referenced publications and https://avdc.gsfc.nasa.gov/pub/data/satellite/Aura/OMI/V03/L2OVP/OMTO3/.

## Code availability

The radiative transfer code TUV is publicly available as described in the references. Graphics and analysis use OriginLab Origin Pro software.
