# Supplementary material for: UVB (290–315 nm) inactivation of the SARS CoV-2 virus as a function of the standard UV index
Source: Air Qual Atmos Health. 2021 Nov 4;15(1):85–90. doi: 10.1007/s11869-021-01099-3 (PMC8567976; doi:10.1007/s11869-021-01099-3)
Supplement: Supplementary file 1 — (DOCX 78 kb) [file 11869_2021_1099_MOESM1_ESM.docx]

**Appendix**

| Table 1 Location, altitude (km) and power law fitting coefficients for T_90_ = a(UVI)^b^ | | | | | | |
| --- | --- | --- | --- | --- | --- | --- |
| Location | Lat | Lon | Alt | a | b |  |
| Abidjan_CL | 5.34 | -4.02 | 0.01 | 49.1970 | -0.9977 |  |
| Abuja_HG | 9.07 | 7.49 | 0.01 | 48.7265 | -0.9881 |  |
| Abu_Dhabi_AE | 24.45 | 54.37 | 0.01 | 58.7409 | -1.0544 |  |
| Accra_GH | 5.56 | -0.19 | 0.03 | 49.2798 | -0.9982 |  |
| Adelaide_AU | -34.92 | 138.6 | 0 | 82.0280 | -1.2080 |  |
| Ahmedabad_IN | 23.03 | 72.59 | 0.06 | 56.7088 | -1.0409 |  |
| Albuquerque_NM | 35.1 | -106.6 | 1.58 | 76.6604 | -1.1557 |  |
| Alexandria_EG | 31.21 | 29.92 | 0 | 71.1310 | -1.1303 |  |
| Algiers_DZ | 36.74 | 3.09 | 0.19 | 81.4606 | -1.1933 |  |
| Alice_Springs_AU | -23.7 | 133.88 | 0.58 | 67.9576 | -1.1290 |  |
| Alta_Floresta_BR | 9.87 | -55.64 | 2.02 | 49.7022 | -1.0055 |  |
| Anchorage_AK_US | 61.1 | -149.9 | 0.03 | 87.4102 | -1.1950 |  |
| Ankara_TR | 39.93 | 32.87 | 0.85 | 86.3545 | -1.2296 |  |
| Annopolis_MD_US | 39.04 | -76.26 | 0 | 80.7872 | -1.1792 |  |
| Aosta_IT | 45.73 | 7.31 | 0.58 | 90.5618 | -1.2700 |  |
| Arica_CL | 18.05 | -70.22 | 0.35 | 51.8967 | -1.0093 |  |
| Athens_GR | 37.98 | 23.73 | 0.72 | 85.4570 | -1.2106 |  |
| Atlanta_GA_US | 33.5 | -84.5 | 0.31 | 71.8574 | -1.1215 |  |
| Auckland_NZ | -36.85 | 174.76 | 0.05 | 85.5233 | -1.2235 |  |
| Baghdad_IQ | 33.34 | 44.4 | 0.04 | 70.9084 | -1.1284 |  |
| Baltimore_US | 39.3 | -76.61 | 0.05 | 81.1263 | -1.1813 |  |
| Bangalore_IN | 12.97 | 77.58 | 0.91 | 50.3355 | -1.0086 |  |
| Bangkok_TH | 13.74 | 100.52 | 0.01 | 50.7754 | -1.0117 |  |
| Bangor_ME | 44.81 | -68.8 | 0.05 | 93.4909 | -1.2558 |  |
| Baoding_CN | 38.85 | 115.49 | 0.02 | 80.9828 | -1.1770 |  |
| Baton_Rouge_US | 30.47 | -91.15 | 0 | 65.2081 | -1.0808 |  |
| Beijing_CN | 39.9 | 116.4 | 0.05 | 84.1552 | -1.1982 |  |
| Belsk_PO | 51.96 | 20.3 | 0.17 | 91.4828 | -1.2438 |  |
| Beltsville_MS_US | 39.03 | -76.76 | 0.03 | 81.1741 | -1.1816 |  |
| Berlin_DE | 52.52 | 13.41 | 0.04 | 91.6423 | -1.2481 |  |
| Bogota_CO | 4.62 | -74.06 | 2.54 | 47.9228 | -1.0017 |  |
| Boston_MA_US | 42.36 | -71.05 | 0.04 | 88.1999 | -1.2229 |  |
| Brasilia_BR | -15.83 | -47.93 | 1.17 | 52.9082 | -1.0322 |  |
| Brisbane_AU | -27.47 | 153.03 | 0.03 | 68.4930 | -1.1237 |  |
| Brussels_BE | 50.8 | 4.35 | 0.1 | 91.6445 | -1.2568 |  |
| Budapest_HU | 47.93 | 20.5 | 0.29 | 96.3297 | -1.2868 |  |
| Buenos_Aires_AR | -34.6 | -58.4 | 0.03 | 72.2125 | -1.1495 |  |
| Bulawayo_ZW | -20.15 | 28.58 | 1.35 | 56.1679 | -1.0488 |  |
| Busan_KR | 35.17 | 129.07 | 0 | 74.6758 | -1.1322 |  |
| Cabauw_NL | 51.82 | 4.59 | 0 | 91.9707 | -1.2524 |  |
| Cairo_EG | 30.06 | 31.25 | 0.02 | 68.1593 | -1.1157 |  |
| Calgary_CA | 51.05 | -114.08 | 1.04 | 90.9325 | -1.2386 |  |
| Canberra_AU | -35.28 | 149.13 | 0.57 | 81.5276 | -1.2032 |  |
| Cape_Town_ZA | -39.92 | 18.42 | 0.04 | 96.0270 | -1.2813 |  |
| Caracas_VZ | 10.5 | -66.92 | 0.9 | 49.5186 | -1.0027 |  |
| Casablanca_MA | 33.59 | -7.6 | 0.03 | 75.3319 | -1.1600 |  |
| Chengdu_CN | 30.67 | 104.07 | 0.5 | 65.1373 | -1.0962 |  |
| Chennai_IN | 13.07 | 80.24 | 0.02 | 50.1463 | -1.0036 |  |
| Chicago_IL_US | 41.89 | -87.67 | 0.19 | 84.4830 | -1.2102 |  |
| Chongqing_CN | 29.56 | 106.55 | 0.22 | 62.3072 | -1.0777 |  |
| Christchurch_NZ | -43.53 | 172.64 | 0.02 | 95.4892 | -1.2987 |  |
| Cordoba_AR | -31.41 | 64.18 | 0.4 | 69.8558 | -1.1383 |  |
| Dallas_TX_US | 32.78 | -96.81 | 0.14 | 68.9655 | -1.1078 |  |
| Darwin_AU | -12.5 | 130.8 | 0.01 | 48.6990 | -0.9970 |  |
| Dar_es_Salaam_TZ | -6.8 | 39.28 | 0.02 | 51.2161 | -1.0220 |  |
| Delhi_IN | 28.65 | 77.23 | 0.23 | 61.3935 | -1.0580 |  |
| Denver_CO_US | 39.74 | -104.96 | 1.64 | 81.2478 | -1.1822 |  |
| Des Moines_IA_US | 41.6 | -93.6 | 0.29 | 83.9589 | -1.2008 |  |
| Detroit_MI_US | 42.3 | -83.05 | 0.19 | 84.8808 | -1.2182 |  |
| Dhaka_BD | 23.7 | 90.4 | 0.01 | 55.8299 | -1.0310 |  |
| Dongguan_CN | 23.02 | 113.75 | 0.01 | 54.4268 | -1.0224 |  |
| Dubai_AE | 25.07 | 55.17 | 0 | 55.2423 | -1.0115 |  |
| Eureka_CA_US | 40.8 | -124.1 | 0.01 | 88.2112 | -1.2316 |  |
| Flagstaff_AZ_US | 35.2 | -111.65 | 2.13 | 79.3781 | -1.1726 |  |
| Giza_EG | 30.01 | 31.21 | 0.03 | 68.0985 | -1.1152 |  |
| Glascow_UK | 55.91 | -3.97 | 0.16 | 90.6942 | -1.2378 |  |
| Greenbelt_MD_US | 39 | -76.9 | 0.06 | 76.6481 | -1.1562 |  |
| Grenada_ES | 37.16 | -3.55 | 0.81 | 85.6221 | -1.2021 |  |
| Griffin_GA_US | 33.2 | -84.28 | 0.29 | 71.5662 | -1.1197 |  |
| Guangzhou_CN | 23.13 | 113.25 | 0.01 | 54.8047 | -1.0261 |  |
| Hamilton_NZ | -37.85 | 175.32 | 0.05 | 86.7452 | -1.2341 |  |
| Hangzhou_CN | 30.29 | 120.16 | 0.01 | 62.5829 | -1.0670 |  |
| Hanoi_VN | 21.02 | 105.84 | 0.01 | 54.2574 | -1.0274 |  |
| Hartford_CT_US | 41.8 | -72.8 | 0.01 | 86.7443 | -1.2098 |  |
| Havana_CU | 23.25 | -82.66 | 0 | 57.7416 | -1.0454 |  |
| Helsinki_FI | 60.17 | 24.94 | 0.01 | 91.6701 | -1.2155 |  |
| Hong_Kong_CN | 22.29 | 114.16 | 0 | 54.0863 | -1.0219 |  |
| Honolulu_HI_US | 21.3 | -157.8 | 0.01 | 59.0532 | -1.0575 |  |
| Houston_TX_US | 29.75 | -95.36 | 0.01 | 66.6127 | -1.0938 |  |
| Ho_Chi_Minh_VN | 10.76 | 106.66 | 0.01 | 48.9565 | -0.9993 |  |
| Hyderabad_IN | 17.36 | 78.46 | 0.51 | 51.2234 | -1.0054 |  |
| Indianapolis_OH_US | 39.77 | -86.16 | 0.25 | 82.6428 | -1.1930 |  |
| Iowa_Center_IA_US | 42 | -93.5 | 0.3 | 82.9343 | -1.1945 |  |
| Iquitos_PE | -3.75 | -73.25 | 0.13 | 48.2942 | -1.0038 |  |
| Ispra_IT | 45.82 | 7.72 | 2.01 | 92.3851 | -1.2740 |  |
| Istanbul_CN | 41.02 | 28.98 | 0.04 | 87.2317 | -1.2281 |  |
| Izania_ES | 28.33 | -16.56 | 1.19 | 68.0895 | -1.1014 |  |
| Jakarta_ID | -6.21 | 106.85 | 0.08 | 49.1018 | -1.0087 |  |
| Jokioinen_FI | 60.8 | 23.48 | 0.12 | 86.3719 | -1.1645 |  |
| Kansas_City_US | 39.09 | -94.57 | 0.29 | 71.6360 | -1.1145 |  |
| Karachi_PK | 24.95 | 67.01 | 0 | 54.2349 | -1.0015 |  |
| Kinshasa_CD | -4.32 | 15.31 | 0.31 | 50.5457 | -1.0134 |  |
| Kislovodsk_RU | 43.91 | 42.72 | 0.81 | 84.9806 | -1.2452 |  |
| Lagos_NG | 6.47 | 3.41 | 0.01 | 49.0924 | -0.9950 |  |
| Lahore_PK | 31.55 | 74.34 | 0.22 | 66.3972 | -1.0920 |  |
| Lamar_CO_US | 38.09 | -102.62 | 1.1 | 76.0983 | -1.1400 |  |
| Lansing_MI_US | 42.73 | -84.56 | 0.03 | 79.4952 | -1.1980 |  |
| Lauder_NZ | -45.05 | 169.7 | 0.37 | 95.8762 | -1.2882 |  |
| La_Paz_BO | -16.5 | -68.15 | 3.78 | 47.9579 | -0.9942 |  |
| La_Quiaca_AR | -22.11 | -65.57 | 4.46 | 71.4837 | -1.1458 |  |
| Leeds_UK | 53.8 | -1.55 | 0.03 | 85.5101 | -1.1906 |  |
| Lima_PE | -12.04 | -77.03 | 0.15 | 56.9655 | -1.0708 |  |
| London_UK | 51.51 | -0.12 | 0.02 | 83.5274 | -1.2054 |  |
| Los_Angeles_CA_US | 34.5 | -118.5 | 0.1 | 76.6976 | -1.1638 |  |
| Madrid_ES | 40.42 | -3.7 | 0.67 | 90.4199 | -1.2233 |  |
| Makassar_ID | -5.13 | 119.4 | 0.01 | 48.2366 | -1.0032 |  |
| Manaus_BR | -3.12 | -60 | 0.09 | 47.8463 | -0.9949 |  |
| Manchester_UK | 53.6 | -1.97 | 0.33 | 87.0487 | -1.2036 |  |
| Manhattan_NY_US | 40.76 | -73.97 | 0.01 | 92.9374 | -1.2339 |  |
| Marimbo_AR | -33.42 | -63.29 | 0.2 | 72.9564 | -1.1529 |  |
| Marin_County_CA_US | 37.5 | -122 | 0.1 | 90.1399 | -1.2151 |  |
| Mauna_Loa_Obs_HI | 19.54 | 155.6 | 3.4 | 55.5015 | -1.0411 |  |
| Melbourne_AU | -37.3 | 145 | 0.01 | 78.3895 | -1.2019 |  |
| Mendoza_AR | -32.9 | -68.9 | 0.83 | 72.1802 | -1.1378 |  |
| Mexico_City_MX | 19.43 | -99.13 | 2.24 | 51.4674 | -1.0064 |  |
| Miami_FL_US | 25.77 | -80.19 | 0.03 | 58.2397 | -1.0413 |  |
| Monterrey_MX | 25.68 | -100.32 | 1.78 | 60.1192 | -1.0570 |  |
| Montreal_CA | 45.45 | -79.93 | 0.02 | 91.0851 | -1.2449 |  |
| Moscow_RU | 55.75 | 37.62 | 0.14 | 87.8258 | -1.1924 |  |
| Mt_Everest_0km | 28 | 86.9 | 0 | 61.8633 | -1.0852 |  |
| Mt_Everest_8.85 | 28 | 86.9 | 8.85 | 62.5004 | -1.0788 |  |
| Mt_Kenya_KE | 0.13 | 37.3 | 5.2 | 48.2012 | -0.9987 |  |
| Mumbai_IN | 19.08 | 72.88 | 0.02 | 51.9319 | -1.0037 |  |
| NAHA_JP | 26.21 | 127.68 | 0.05 | 57.5620 | -1.0466 |  |
| Nairobi_KE | 1.09 | 35.88 | 1.86 | 48.5068 | -1.0020 |  |
| Nanjing_CN | 32.06 | 118.78 | 0.02 | 62.6556 | -1.0548 |  |
| New_Delhi_IN | 28.61 | 77.2 | 0.03 | 65.2451 | -1.0921 |  |
| New_Orleans_US | 29.95 | -90.08 | 0.02 | 64.7008 | -1.0763 |  |
| New_York_US | 40.71 | -71.01 | 0.06 | 82.9985 | -1.1948 |  |
| Nice_FR | 43.67 | 7.29 | 0.03 | 100.6360 | -1.2772 |  |
| Obninsk_RU | 55.1 | 36.61 | 0.17 | 86.4005 | -1.1864 |  |
| OceanA00E | 0 | -30 | 0 | 48.8068 | -1.0002 |  |
| OceanA05N | 5 | -30 | 0 | 49.2795 | -1.0047 |  |
| OceanA05S | -5 | -30 | 0 | 50.5021 | -1.0122 |  |
| OceanA10N | 10 | -30 | 0 | 50.9441 | -1.0133 |  |
| OceanA10S | -10 | -30 | 0 | 52.7336 | -1.0277 |  |
| OceanA15N | 15 | -30 | 0 | 57.0174 | -1.0532 |  |
| OceanA15S | -15 | -30 | 0 | 54.2269 | -1.0362 |  |
| OceanA20N | 20 | -30 | 0 | 74.8476 | -1.1628 |  |
| OceanA20S | -20 | -30 | 0 | 58.1168 | -1.0613 |  |
| OceanA25N | 25 | -30 | 0 | 81.1322 | -1.1897 |  |
| OceanA25S | -25 | -30 | 0 | 61.9373 | -1.0841 |  |
| OceanA30N | 30 | -30 | 0 | 86.6993 | -1.2140 |  |
| OceanA30S | -30 | -30 | 0 | 67.7177 | -1.1194 |  |
| OceanA35N | 35 | -30 | 0 | 90.0172 | -1.2281 |  |
| OceanA35S | -35 | -30 | 0 | 80.4584 | -1.1883 |  |
| OceanA40N | 40 | -30 | 0 | 90.2386 | -1.2343 |  |
| OceanA40S | -40 | -30 | 0 | 91.6784 | -1.2543 |  |
| OceanA45N | 45 | -30 | 0 | 94.7744 | -1.2730 |  |
| OceanA45S | -45 | -30 | 0 | 97.7396 | -1.3001 |  |
| OceanA50N | 50 | -30 | 0 | 94.0193 | -1.2721 |  |
| OceanA50S | -50 | -30 | 0 | 95.4641 | -1.3102 |  |
| OceanA55N | 55 | -30 | 0 | 97.0243 | -1.2771 |  |
| OceanA55S | -55 | -30 | 0 | 91.2986 | -1.2827 |  |
| OceanA60N | 60 | -30 | 0 | 92.6051 | -1.2295 |  |
| OceanA60S | -60 | -30 | 0 | 74.2106 | -1.1338 |  |
| OceanP00E | 0 | -179 | 0 | 47.5793 | -0.9960 |  |
| OceanP05N | 5 | -179 | 0 | 48.5491 | -1.0051 |  |
| OceanP05S | -5 | -179 | 0 | 48.8476 | -1.0076 |  |
| OceanP10N | 10 | -179 | 0 | 50.2009 | -1.0121 |  |
| OceanP10S | -10 | -179 | 0 | 49.9517 | -1.0169 |  |
| OceanP15N | 15 | -179 | 0 | 56.5131 | -1.0509 |  |
| OceanP15S | -15 | -179 | 0 | 52.6986 | -1.0327 |  |
| OceanP20N | 20 | -179 | 0 | 58.6379 | -1.0565 |  |
| OceanP20S | -20 | -179 | 0 | 58.7785 | -1.0696 |  |
| OceanP25N | 25 | -179 | 0 | 66.2230 | -1.0999 |  |
| OceanP25S | -15 | -179 | 0 | 53.0059 | -1.0343 |  |
| OceanP30N | 30 | -179 | 0 | 73.7596 | -1.1429 |  |
| OceanP30S | -30 | -179 | 0 | 79.0343 | -1.1784 |  |
| OceanP35N | 35 | -179 | 0 | 79.2575 | -1.1728 |  |
| OceanP35S | -35 | -179 | 0 | 87.9778 | -1.2219 |  |
| OceanP40N | 40 | -179 | 0 | 91.7513 | -1.2620 |  |
| OceanP40S | -40 | -179 | 0 | 101.5288 | -1.2948 |  |
| OceanP45N | 45 | -179 | 0 | 106.0718 | -1.3774 |  |
| OceanP45S | -45 | -179 | 0 | 108.9511 | -1.3412 |  |
| OceanP50N | 50 | -179 | 0 | 102.1949 | -1.3544 |  |
| OceanP50S | -50 | -179 | 0 | 105.5614 | -1.3397 |  |
| OceanP55N | 55 | -179 | 0 | 102.8672 | -1.3565 |  |
| OceanP55S | -55 | -179 | 0 | 96.7894 | -1.2963 |  |
| OceanP60N | 60 | -179 | 0 | 100.2197 | -1.3104 |  |
| OceanP60S | -60 | -179 | 0 | 96.1967 | -1.2882 |  |
| Palembang_ID | -2.99 | 104.76 | 0.01 | 49.4721 | -1.0134 |  |
| Paris_FR | 48.86 | 2.35 | 0.04 | 102.6756 | -1.3086 |  |
| Perth_AU | -31.95 | 115.9 | 0.03 | 91.7096 | -1.2405 |  |
| Phoenix_US | 33.45 | -112.07 | 0.37 | 98.0184 | -1.2609 |  |
| Pilar_AR | -31.66 | -63.88 | 0.34 | 81.3701 | -1.1874 |  |
| Portland_US | 45.52 | -122.67 | 0.01 | 95.3607 | -1.2579 |  |
| Punta_Arenas_CL | -53.16 | -70.92 | 0.04 | 99.5985 | -1.3182 |  |
| Quanzhou_CN | 24.91 | 116.59 | 0.01 | 56.1332 | -1.0329 |  |
| Queenstown_SA | -31.9 | 26.92 | 1.1 | 83.0018 | -1.1970 |  |
| Quezon_City_PH | 14.65 | 121.05 | 0.05 | 52.1824 | -1.0256 |  |
| Quito_EC | 0.18 | -78.5 | 2.85 | 47.6888 | -1.0024 |  |
| Recife_BR | -8.05 | -34.93 | 0.55 | 54.0084 | -1.0385 |  |
| Redding_CA_US | 40.5 | -122.4 | 0.03 | 95.9917 | -1.2422 |  |
| Rio_de_Janeiro_BR | -22.91 | -43.2 | 0.05 | 62.0415 | -1.0870 |  |
| Riyadh_SA | 24.77 | 46.74 | 0.61 | 74.8011 | -1.1541 |  |
| Rome_IT | 41.9 | 12.5 | 0.01 | 102.4364 | -1.2799 |  |
| Rosario_AR | -32.94 | -60.64 | 0.03 | 75.9495 | -1.1529 |  |
| Rural_Georgia_GA | 34.5 | -83.5 | 0.2 | 77.5521 | -1.1487 |  |
| S-America00E | 0 | -60 | 0.2 | 48.5000 | -1.0032 |  |
| S-America05N | 5 | -60 | 1 | 49.6582 | -1.0118 |  |
| S-America05S | -5 | -60 | 2 | 47.6112 | -0.9926 |  |
| S-America10N | 10 | -60 | 0 | 52.1703 | -1.0254 |  |
| S-America10S | -10 | -60 | 0.2 | 48.2227 | -0.9938 |  |
| S-America15N | 15 | -60 | 0 | 57.0207 | -1.0534 |  |
| S-America15S | -15 | -60 | 0.2 | 50.7440 | -1.0121 |  |
| S-America20N | 20 | -60 | 0 | 58.3915 | -1.0540 |  |
| S-America20S | -20 | -60 | 0.05 | 54.2903 | -1.0352 |  |
| S-America25N | 25 | -60 | 0 | 57.8697 | -1.0366 |  |
| S-America25S | -25 | -60 | 0.05 | 60.3593 | -1.0785 |  |
| S-America30N | 30 | -60 | 0 | 67.4278 | -1.0994 |  |
| S-America30S | -30 | -60 | 0.05 | 69.4836 | -1.1239 |  |
| S-America35N | 35 | -60 | 0 | 76.0658 | -1.1470 |  |
| S-America35S | -35 | -60 | 0.05 | 75.1879 | -1.1598 |  |
| S-America40N | 40 | -60 | 0 | 81.7357 | -1.1859 |  |
| S-America40S | -40 | -60 | 0 | 93.5147 | -1.2452 |  |
| S-America45N | 45 | -60 | 0 | 87.6791 | -1.2091 |  |
| S-America45S | -45 | -60 | 0 | 89.2461 | -1.2460 |  |
| S-America50N | 50 | -60 | 0 | 87.2079 | -1.2137 |  |
| S-America50S | -50 | -60 | 0 | 83.3225 | -1.2020 |  |
| S-America55N | 55 | -60 | 0 | 80.0440 | -1.1451 |  |
| S-America55S | -55 | -60 | 0 | 81.7372 | -1.2060 |  |
| S-America60N | 60 | -60 | 0.5 | 88.1614 | -1.1843 |  |
| S-America60S | -60 | -60 | 0 | 79.1337 | -1.1757 |  |
| Sacramento_CA_US | 38.5 | -121.5 | 0.08 | 83.7081 | -1.1886 |  |
| Saint_Petersburg_RU | 59.98 | 30.32 | 0.01 | 85.0982 | -1.1580 |  |
| Salt_Lake_UT_US | 40.7 | -111.9 | 1.32 | 84.3226 | -1.2137 |  |
| Salvador_BR | -12.97 | -38.48 | 0.01 | 53.2326 | -1.0374 |  |
| San Diego_CA_US | 32.77 | 117.19 | 0.01 | 65.3570 | -1.0785 |  |
| Santa FE_NM_US | 35.69 | -105.94 | 2.14 | 77.7688 | -1.1682 |  |
| Santa_Rosa_CA_US | 38.5 | -122.7 | 0.05 | 91.6325 | -1.2249 |  |
| Santiago_CL | -33.46 | -70.65 | 0.56 | 67.7512 | -1.1259 |  |
| San_Antonio_TX_US | 29.42 | -98.49 | 0.2 | 63.3581 | -1.0748 |  |
| San_Francisco_US | 37.77 | -122.42 | 0.03 | 85.0888 | -1.2127 |  |
| San_Jose_CA_US | 37.5 | -122.5 | 0.14 | 81.4059 | -1.1897 |  |
| San_Julian_AR | -49.32 | -67.75 | 0.06 | 92.4521 | -1.2633 |  |
| San_Pedro_CL | -22.9 | -68.2 | 2.45 | 67.0313 | -1.1184 |  |
| Sao Paulo_BR | -23.55 | -46.64 | 0.77 | 59.9921 | -1.0764 |  |
| Sapporo_JP | 43.08 | 140.76 | 0.36 | 85.0696 | -1.2051 |  |
| Seattle_WA_US | 47.5 | -123.5 | 0.14 | 90.9241 | -1.2394 |  |
| Seoul_KR | 37.56 | 126.98 | 0.04 | 78.3848 | -1.1560 |  |
| Shanghai_CN | 31.22 | 121.47 | 0.06 | 65.2016 | -1.0790 |  |
| Shenyang_CN | 41.79 | 123.43 | 0.05 | 103.4311 | -1.3020 |  |
| Shenzhen_CN | 22.55 | 114.07 | 0 | 53.2756 | -1.0112 |  |
| Singapore_SG | 1.29 | 103.85 | 0.02 | 48.7652 | -1.0075 |  |
| Sodankyla_FI | 67.42 | 26.6 | 0.18 | 95.9325 | -1.2212 |  |
| Stanley_FK | -51.7 | -57.9 | 0.05 | 93.9208 | -1.2735 |  |
| Steamboat_Spr_US | 40.48 | -106.83 | 2.07 | 81.5384 | -1.1934 |  |
| St_Louis_MO_US | 38.63 | -90.2 | 0.15 | 78.8462 | -1.1639 |  |
| Suzhou_CN | 31.3 | 120.6 | 0.01 | 60.6196 | -1.0351 |  |
| Tampa_FL_US | 28 | -82.5 | 0.01 | 59.2419 | -1.0390 |  |
| Tehran_IR | 35.69 | 51.42 | 1.18 | 75.6110 | -1.1473 |  |
| Tel-Aviv_IL | 32.11 | 34.86 | 0.03 | 76.1470 | -1.1530 |  |
| Tianjin_CN | 39.14 | 117.18 | 0.01 | 90.6642 | -1.2272 |  |
| Tokyo_JP | 35.65 | 139.84 | 0.04 | 68.2208 | -1.0840 |  |
| Toronto_CA | 43.65 | -79.35 | 0.17 | 92.5215 | -1.2472 |  |
| Tuscon_AZ_US | 32.22 | -110.3 | 0.76 | 73.7889 | -1.1321 |  |
| Ushuaia_AR | -54.8 | -68.3 | 0.06 | 88.0116 | -1.2544 |  |
| Utah_Center_UT_US | 39 | -109.5 | 1.8 | 81.0316 | -1.1705 |  |
| Vancouver_CA | 49.24 | -123.12 | 0.07 | 87.8248 | -1.2116 |  |
| Vientiane_LA | 17.97 | 102.63 | 0.17 | 52.2888 | -1.0159 |  |
| Waimea_HA_US | 21.96 | -159.68 | 0.01 | 63.6462 | -1.0918 |  |
| Washington_DC_US | 38.9 | -77.04 | 0.01 | 80.9875 | -1.1843 |  |
| Wellington_NZ | -41.3 | 174.8 | 0.08 | 89.2575 | -1.2309 |  |
| Wenzhou_CN | 28 | 120.67 | 0.01 | 58.4668 | -1.0435 |  |
| White_Sands_NM | 32.4 | -106.5 | 1.22 | 73.7651 | -1.1317 |  |
| Wuhan_CN | 30.58 | 114.27 | 0.02 | 62.8057 | -1.0745 |  |
| Yangon_MM | 30.58 | 114.27 | 0.02 | 62.3861 | -1.0713 |  |
| Zugspitze_DE | 30.58 | 114.27 | 0.02 | 61.9639 | -1.0680 |  |

| Table 2 Coefficients for the series in Eqn. A1 for Fig. 5.  −60^O^ ≤ θ ≤ 60^O^ and t = 12:00. | | | | |
| --- | --- | --- | --- | --- |
| T_90_(θ,UVI) = c_0_ + c_1_θ + c_2_θ^2^ + c_3_θ^3^ + c_4_θ^4^ + c_5_θ^5^ +c_6_θ^6^ | | | | |
| Coef | UVI=6 | UVI=8 | UVI=10 | UVI=12 |
| c_0_ | 8.0581E+00 | 6.0777E+00 | 4.8813E+00 | 4.0797E+00 |
| c_1_ | -7.8426E-04 | 1.1600E-03 | 1.9600E-03 | 2.3100E-03 |
| c_2_ | 1.8700E-03 | 8.9046E-04 | 4.0993E-04 | 1.4368E-04 |
| c3 | 3.0060E-06 | 2.2329E-06 | 1.7424E-06 | 1.4075E-06 |
| c_4_ | -6.6975E-07 | -3.0064E-07 | -1.2115E-07 | -2.2702E-08 |
| c_5_ | -3.3575E-10 | -4.0633E-10 | -4.0770E-10 | -3.8945E-10 |
| c_6_ | 9.5669E-11 | 5.0616E-11 | 2.7885E-11 | 1.4920E-11 |

|  | **A1** |
| --- | --- |

| Table 3 Coefficients for the series in Eqn. A1 for Fig. 6a.  −60^O^ ≤ θ ≤ 60^O^ and t = 13:00. | | | | |
| --- | --- | --- | --- | --- |
| T_90_(θ,UVI) = c_0_ + c_1_θ + c_2_θ^2^ + c_3_θ^3^ + c_4_θ^4^ + c_5_θ^5^ +c_6_θ^6^ | | | | |
| Coef | UVI=6 | UVI=8 | UVI=10 | UVI=12 |
| c_0_ | 8.2676E+00 | 6.2295E+00 | 4.9996E+00 | 4.1762E+00 |
| c_1_ | -1.7200E-03 | 1.5500E-03 | 2.9200E-03 | 3.5200E-03 |
| c_2_ | 1.8100E-03 | 8.1501E-04 | 3.3013E-04 | 6.4606E-05 |
| c3 | 3.4691E-06 | 1.2199E-06 | 1.4186E-07 | -4.3659E-07 |
| c_4_ | -6.7757E-07 | -2.7079E-07 | -7.6601E-08 | 2.7770E-08 |
| c_5_ | -3.3328E-10 | -6.9452E-11 | 5.6992E-11 | 1.2412E-10 |
| c_6_ | 1.0230E-10 | 4.8629E-11 | 2.2276E-11 | 7.6570E-12 |

| Table 4 Coefficients for the series in Eqn. A1 for Fig. 6b.  −60^O^ ≤ θ ≤ 60^O^ and t = 14:00 | | | | |
| --- | --- | --- | --- | --- |
| T_90_(θ,UVI) = c_0_ + c_1_θ + c_2_θ^2^ + c_3_θ^3^ + c_4_θ^4^ + c_5_θ^5^ +c_6_θ^6^ | | | | |
| Coef | UVI=6 | UVI=8 | UVI=10 | UVI=12 |
| c_0_ | 9.0328E+00 | 6.7764E+00 | 5.4209E+00 | 4.5165E+00 |
| c_1_ | 1.8000E-03 | 4.3500E-03 | 5.2300E-03 | 5.4900E-03 |
| c_2_ | 1.1800E-03 | 3.0826E-04 | -9.0232E-05 | -2.9489E-04 |
| c3 | 2.4618E-06 | 4.7818E-07 | -4.3011E-07 | -8.9203E-07 |
| c_4_ | -3.7150E-07 | 6.9727E-10 | 1.6444E-07 | 2.4378E-07 |
| c_5_ | -2.4975E-10 | -1.5573E-11 | 9.2441E-11 | 1.4726E-10 |
| c_6_ | 6.2379E-11 | 9.9656E-12 | -1.3641E-11 | -2.5453E-11 |
